# Free-Riding Behavior in Vaccination Decisions: An Experimental Study

**DOI:** 10.1371/journal.pone.0087164

**Published:** 2014-01-24

**Authors:** Yoko Ibuka, Meng Li, Jeffrey Vietri, Gretchen B. Chapman, Alison P. Galvani

**Affiliations:** 1 Graduate School of Economics, Tohoku University, Sendai, Miyagi, Japan; 2 Department of Health and Behavioral Sciences, University of Colorado Denver, Denver, Colorado, United States of America; 3 Health Economics and Outcomes Research, Kantar Health, Princeton, New Jersey, United States of America; 4 Department of Psychology, Rutgers University, Piscataway, New Jersey, United States of America; 5 Department of Epidemiology and Public Health, Yale University School of Medicine, New Haven, Connecticut, United States of America; Tohoku University, Japan

## Abstract

Individual decision-making regarding vaccination may be affected by the vaccination choices of others. As vaccination produces externalities reducing transmission of a disease, it can provide an incentive for individuals to be free-riders who benefit from the vaccination of others while avoiding the cost of vaccination. This study examined an individual's decision about vaccination in a group setting for a hypothetical disease that is called “influenza” using a computerized experimental game. In the game, interactions with others are allowed. We found that higher observed vaccination rate within the group during the previous round of the game decreased the likelihood of an individual's vaccination acceptance, indicating the existence of free-riding behavior. The free-riding behavior was observed regardless of parameter conditions on the characteristics of the influenza and vaccine. We also found that other predictors of vaccination uptake included an individual's own influenza exposure in previous rounds increasing the likelihood of vaccination acceptance, consistent with existing empirical studies. Influenza prevalence among other group members during the previous round did not have a statistically significant effect on vaccination acceptance in the current round once vaccination rate in the previous round was controlled for.

## Introduction

Promoting vaccination is an important goal in public health policy. However, influenza vaccination coverage in the United States is still far below the public policy goal. Vaccination may be discouraged by the incentive to “free-ride”. Referred to herein as “free-riders” in vaccination, these individuals avoid the cost associated with vaccination while benefiting from other individuals' vaccination [1] [2]. Vaccination for infectious diseases produces herd immunity, providing indirect benefit to unvaccinated individuals. As the result of herd immunity, the risk of infection for an individual depends on other individuals' vaccination status; risk of infection generally decreases as the vaccination coverage in a community increases regardless of an individual's vaccination status.

In economic theory, a free-riding problem occurs in the market of public goods that have two main characteristics: non-rivalry and non-exclusion of consumption. Vaccination holds both characteristics. Non-rival consumption indicates that consumption of a good by one person does not affect the quantities consumed by other individuals. Goods involving non-exclusion are costly or sometimes impossible to restrict their benefits to certain individuals. The production of public goods results in positive externalities, and accordingly, herd immunity effects are described as positive externalities. Vaccination externalities have been theoretically analyzed in both a static [3] [4] and a dynamic framework [4] [5] [6].

As empirical analyses, previous survey studies have demonstrated some evidence of free-riding in vaccination decision-making. Based on hypothetical scenarios regarding vaccination against an infectious disease, free-riding incentives have been found to significantly influence vaccination decisions [7]. In another study on parents' vaccination choice, parents answered that they would be less likely to vaccinate their children if most of their children's contacts were vaccinated [8], a response pattern that is consistent with free-riding.

In this study, we used a computerized interactive game to examine individuals' decision making about vaccination for a simulated influenza infection in a group setting. Specifically, we determined the role of free-riding incentives as well as other potential factors in vaccination decision-making. Due to uncertainty regarding the outcome, the decision to get vaccinated is dependent on more factors than ordinary economic goods, including health status, individuals' beliefs, and other psychological factors such as fears or regrets [9], [10], [11], [12], [13]. We attempted to examine how individuals make a decision in response to observed decisions made by other individuals, controlling for these effects using experimental approach.

In the game, eight to ten participants formed a group. Participants simultaneously and independently made a decision on whether they would get vaccinated given a set of parameter conditions specific to each round, sequentially playing 24 rounds of the game. Each participant was granted 2,000 points initially and lost points when they “spent” points on buying the vaccine or when they were infected. Final points were connected to monetary payouts for participants. In addition to free-riding incentives, we examined whether an individual's influenza exposure in prior rounds of the game or the influenza prevalence in the group would influence further decisions about vaccination acceptance. The experimental design mirrored the dynamic nature of influenza transmission where individuals' chance of “infection” depended on the realized proportion of vaccination in the group. Our results showed that individuals' vaccination decisions were significantly influenced by observed vaccination rate during the previous round, suggesting free-riding behavior.

## Methods

### Experimental Design

This study was approved by Rutgers University Institutional Review Board. In the experiment, participants provided assent in the following way. The consent form appeared on the computer screen when participants first sat down, and they read the form and then advanced to the next screen only if they wished to participate in the study. Thus, the fact that they provided responses on later computer screens documented that they consented to participant. As the research was low risk, the Institutional Review Board ruled that signatures on consent forms were not necessary.

The computerized game experiment was conducted between April and October in 2008 with 269 undergraduate students at Rutgers University. The experiment assumed a fictional scenario for influenza vaccination, and in each round participants were required to decide whether they would get vaccinated against the influenza under a certain set of conditions controlled by parameters in the experiment.

Eight to ten participants formed one group and played 24 rounds within the group. Each round ran independently and thus participants did not carry immunity over different rounds of the game. Each player started initially with 2,000 points, but lost points when they were vaccinated due to the cost of the vaccine, and more points if they were infected due to the costs of infection. The probability of infection was assumed to depend both on the proportion of vaccination in the group and on each individual's vaccination status (Table 1). Getting vaccinated reduced an individual's risk of infection by half. To provide participants with an incentive, the final points at the end of the game were translated into real monetary payouts ranging from zero to ten dollars. The conversion rate was 2 point  = 1 cent. The instructions to participants indicated that the 24 rounds could be considered as 24 influenza seasons.

**Table 1:** Parameter values in the experimental game: transmissibility table that shows the relationship between the proportion of vaccination and risk of infection.

	[A] Low Risk Condition		[B] High Risk Condition	
Percent of players who get vaccinated	Without vaccination	With vaccination	Without vaccination	With vaccination
0	80	40	100	50
10	70	35	100	50
20	10	5	100	50
30	5	3	100	50
40	1	1	90	45
50	0	0	80	40
60	0	0	70	35
70	0	0	10	5
80	0	0	5	3
90	0	0	1	1
100	0	0	0	0

The experiment consisted of three structural levels: group, player and round. Among the three levels, the instruction conditions were designed for another study and not directly relevant to the purpose of our study. Therefore we will not further mention the details about the instruction conditions in the rest of the paper although we controlled for the instruction conditions in all the regression analyses.

At the player level, *player type* was varied among players within each group. Each player was randomly assigned to play either as a *young* person or as an *elderly* person at the beginning of the game with fifty percent probability. When infected, elderly players lost more points than young players, thus mirroring the more severe real-life consequences of influenza infection in elderly individuals than in young individuals. The distinction of young versus elderly varied between players but was fixed for a given player across the rounds.

Three parameters were assigned in each round (Table 2): (a) *cost of vaccine* (*high cost* or *low cost*); (b) *risk of infection* (*high risk* or *low risk*); and (c) *severity of influenza* (*severe* or *mild*). The 24 rounds in a game consisted of three sequential repetitions of each combination of the three parameters (2×2×2 with three replications). One set of parameters was given and maintained within a group during three consecutive rounds. The order of the parameter combinations was randomly assigned to each group. The cost was 60 points for the *high cost* of vaccine condition and 20 points for the *low cost* of vaccine condition. Under s*evere* disease outbreak conditions, the young players lost 100 points and elderly players lost 400 points if they were not vaccinated and were infected with the influenza. By contrast, under *mild* disease outbreaks, *young* players lost 100 points, whereas *elderly* players lost 150 points if they were not vaccinated and were infected. If players were vaccinated and infected with the influenza, they lost half of the points they would have lost without vaccination (Table 2). The risk of infection was also varied between two risk conditions and determined by vaccination coverage in the group (Table 1). In the experiment, an epidemic stops once vaccination reaches a threshold level in the community, mirroring the epidemiology of infectious diseases [14].

**Table 2:** Parameter values in the experimental game: initial points, cost of vaccine, and severity of influenza.

Initial points	Cost of vaccine		Severity of influenza (without/with vaccination)		
2,000	Low	20	Young	Severe	100/50
	High	60		Mild	100/50
			Elderly	Severe	400/200
				Mild	150/75

Participants were seated at computer cubicles. Before starting the computerized experiment, the participants viewed an instructional slide show that explained the design of the experiment including the role of young and elderly players and how transmission varies with vaccination rates using the same table as Table 1. Also, at the end of each round, each player was notified (i) if they were infected with the influenza and how many total points remained; (ii) the number of players that chose to be vaccinated and how many were infected among the vaccinated and among the unvaccinated players; and (iii) the breakdown of young and elderly players among those who contracted the influenza.

Our observations consisted of 6,456 data points from 269 players, which corresponded to a total of 29 groups. Seven groups consisted of eight players, another seven groups consisted of 9 nine players and 15 groups consisted of 10 players. The instructions took 15 to 20 minutes and the entire game took 20 to 30 minutes for the 24 rounds. The average earning of the all the participants was 7.6 dollars.

### Statistical analysis

To analyze the observations, we used both descriptive statistics and logistic regressions. Our analysis consisted of the following four steps. First, we analyzed the observations with descriptive statistics to examine the relationship between the proportion of vaccination and the parameters in the experiment. Second, we examined the free-riding behavior using multilevel logistic regressions to evaluate the effect of vaccination rate during the previous round on each player's subsequent vaccination decision. Additionally, we examined whether the free-riding behavior varied across different parameter conditions in the experiment. Finally, other potential determinants on vaccination decision-making were evaluated.

To relate the variables of interest with the choice of vaccination, logistic regressions were used. The game involved two sets of nested structure: rounds nested within players and players nested within groups. To analyze the observations while considering the nested structure, multilevel modeling, also known as hierarchical modeling, was employed [15] [16]. The general econometric specification was expressed as follows:
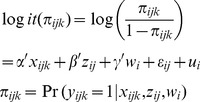
(1),where 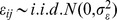
and, 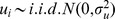 that represent random effects at the player level and at the group level respectively. 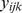 is a dichotomous variable to represent whether participants chose to be vaccinated (yes = 1), 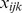 is the column vector that contains independent variables at the round level, 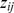 is the column vector that contains independent variables at the player level and 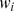 is the column vector that contains the independent variables at the group level. 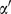, 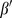 and 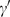 are row vectors that contain the coefficients of the independent variables at the round, player and group level respectively.

To evaluate whether a player's decision about vaccination was influenced by the vaccination decisions of others, we related the proportion of vaccination among others to players' vaccination decisions using equation (1). In each round, all the players made a simultaneous decision and therefore other players' decisions were not observable until all the players made decisions in the same round. Therefore, we hypothesized that players inferred others' decisions based on the outcome of the previous round, and we included the proportion of vaccination among other group members during the previous round in 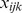, in equation (1) to test free-riding behavior, defined as lower likelihood of vaccination when more players were vaccinated in the previous round.

The regression was controlled by the five parameters in the experiment and a variable ranging from one to 24 that shows the number of rounds played by an individual. Specifically, we included three dichotomous variables to represent the cost of the vaccine (high cost  = 1), the risk of the infection (low risk  = 1) and the severity of influenza (mild condition  = 1) as well as the number of rounds in 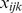; and a binary to represent the player type in 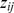 (elderly  = 1).

Next, we added the interaction term between vaccination rate among other group members in the previous round and each of the binary parameters in the experiment to examine whether free-riding varied across different parameter conditions. Interaction terms were added one at a time, providing the total of four regressions. In regressions with interaction terms, the proportion of others vaccinated was centered on the mean to make the coefficient of the interaction term interpretable [17]. Furthermore, we conducted sub-sample analysis, dividing the sample by parameter condition, in order to examine the marginal effect of the proportion of vaccinated on the probability of vaccination acceptance in each parameter condition.

Finally, two other potential factors that may influence vaccination decision-making were assessed: influenza prevalence in the group in the previous round, and an individual's influenza exposure during the game up to the current round. To evaluate the effect of these two factors on vaccination decision, two variables were additionally included in, 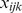 in equation (1). Specifically, the proportion of those infected among other group members during the previous round was used as a measure of the disease prevalence in the group. The number of the player's past infections during the game was used to express players' influenza exposure during the game.

All the regression analyses were performed using Stata 12 (StataCorp LP). Our analysis revealed that the random effect at the group level 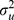, was estimated as zero, indicating that there was no significant random variation among groups beyond the fixed effect included in the model. Therefore, our final model included only the random intercept at the player level. Only observations between round 2 and 24 were used for the analysis, whenever the analysis involved the proportion of vaccination or of those infected during the previous round, as there was no information available for players on the proportion of vaccination during the previous round in the first round.

## Results

### The proportion of vaccination

**Figure 1:**
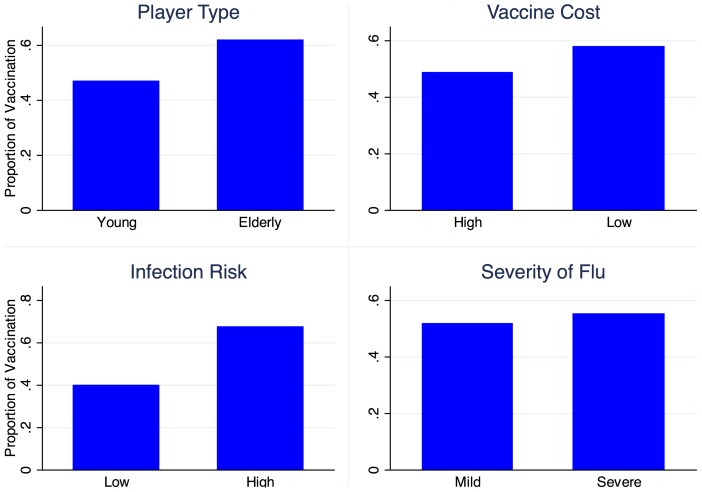
Proportion of vaccination, by parameter condition. All the differences were statistically significant at the 0.05 level evaluated by the odds ratio (OR). The ORs were calculated by the multilevel logistic regression (Eq.1), where 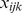 includes the binaries to represent the cost of the vaccine, the risk of infection and the severity of the influenza. 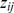 includes the binary to represent players' type, and 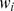 is the group level effect.

The overall vaccination rate was 53%. There was greater vaccination among elderly players compared to young players (62% vs. 47%), when the cost of vaccination was low versus high (58% vs. 49%), when the risk of infection was high versus low (67% vs.40%), and when the influenza was severe versus mild (55% vs. 51%) (Figure 1). The 95 percent confidence intervals of the odds ratios did not include unity, demonstrating significant differences in the probability of vaccination acceptance by the experimental parameters (see Table 3).

**Table 3:** Test for free-riding behavior, results from multilevel logistic regressions.

	Reference group	Coefficient estimate	Standard error	Marginal effect
Proportion of others vaccinated		−0.910^***^	0.17	−0.19
Intercept		1.04^***^	0.17	N.A.
Player type	Young	0.759^***^	0.14	0.16
Cost of vaccine	High	0.559^***^	0.06	0.12
Risk of infection	Low	1.599^***^	0.07	0.36
Severity of the influenza	Mild	0.240^***^	0.06	0.05
Number of rounds		−0.006	0.005	−0.001
Random effect (Player level) a		1.049	0.06	N.A.

* P<0.05, ^**^ P<0.01, ^***^P<0.001

a No formal statistical test was conducted to test if the random effect equals to zero.

Observations from Round 2 to Round 24 were used for the analysis. The random effect at the group level was not shown as it was estimated as zero.

**Figure 2:**
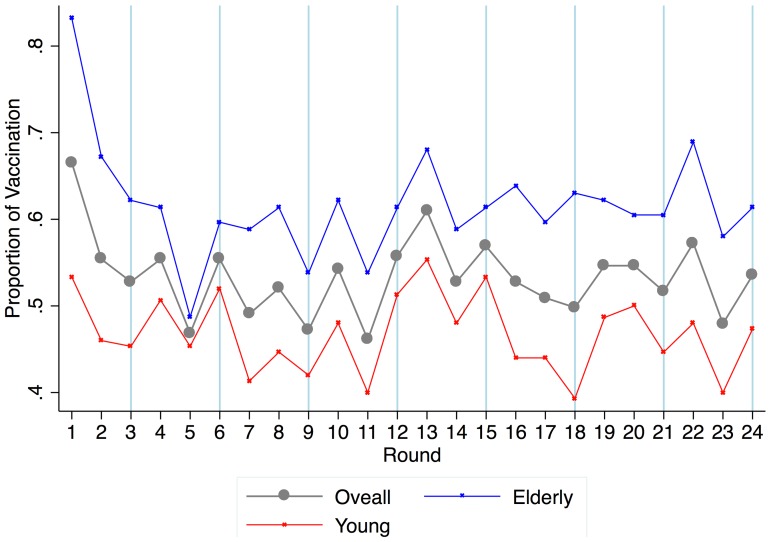
Proportion of vaccination in 24 rounds, by player type. 24 rounds are blocked every three rounds within which the combination of the parameters were constant.

To assess how the proportion of vaccination changed over time, the proportion of those vaccinated during 24 rounds was calculated (Figure 2). The game started with higher overall vaccination rate of 67 percent and dropped by an average of 14 percent in the subsequent two rounds, with a greater drop of 16 percent for the elderly and 7 percent for the young.

### Evidence of Free-riding Behavior

To evaluate how a player's vaccination decision was influenced by other players' vaccination decision-making, we related the proportion of other group members who got vaccinated during the previous round to any one individual's vaccination decision in the current round, using equation (1). The estimated coefficient of the proportion of vaccination was negative and statistically significant, indicating that a player's likelihood to vaccinate lowered with the observed higher vaccination rate (Table 3). Overall, the probability of vaccination decreased by 19 percentage point as the proportion of previous round vaccination among others increased from 0 to 1.

All other estimates for the coefficients showed the expected sign and were statistically significant. The linear trend term of round number was not significant after experimental parameters were controlled for.

### Parameters in the experiment and free-riding

To identify how the result on free-riding behavior would change by the controlled parameters in the experiment, we conducted a regression analysis by equation (1) including interaction terms between each binary experimental parameter and the proportion of others vaccinated in addition to the original set of independent variables. The exponent of coefficient of the interaction term represents the difference between the alternative and reference condition of the binary parameter, in terms of odds per 1-unit increase in the vaccination proportions. If the proportion of others vaccinated has the same impact on the predicted odds between two categories, the coefficient of the interaction term is zero.

Table 4 shows only the results with significant interaction effect. It is also important to notice that the coefficient for the proportion of others vaccinated has a different interpretation from the one in the regressions without interaction terms. It no longer implies the multiplicative factor of the variable for the entire observations, and rather represents the multiplicative factor for the reference group on the parameter in the interaction term. For instance, in the regression that includes the interaction term between the proportion of others vaccinated and the risk of infection, the exponent of the coefficient for the proportion of others vaccinated represents the degree of change in the predicted odds given a 1-unit of increase in the proportion of others vaccinated for those under low risk condition.

**Table 4:** Parameters and free-riding behavior: multilevel logistic regression analysis with an interaction term between the risk of infection parameter and proportion of others vaccinated.

	Reference group	Estimate for logistic coefficient	Exponent of coefficient
Proportion of others vaccinated		−0.518*	0.60
Risk of infection * Proportion of others vaccinated		−0.816*	0.44
Intercept		−1.086	0.34
Player type	Young	0.761^***^	2.14
Cost of vaccine	High	0.557^***^	1.75
Risk of infection	Low	1.993^***^	7.34
Severity of the influenza	Mild	0.232^***^	1.26
Number of rounds		−0.005	1.00
Random effect (Player level) a		1.049	N.A.

* P<0.05, ^**^P<0.01, ^***^P<0.001

a No formal statistical test was conducted to test if the random effect equals zero.

Observations from Round 2 to Round 24 were used for the analysis. The random effect at the group level was not shown as it was estimated as zero.

There was no significant interaction between the proportion of others vaccinated and each binary parameter for all parameters except for risk of infection. This significant interaction term indicates that the multiplicative factor was greater in the high risk condition, suggesting higher impact of the proportion vaccinated on the predicted odds (Table 4). That is, there was a larger degree of free-riding behavior in the high risk condition than in the low risk condition.

**Figure 3:**
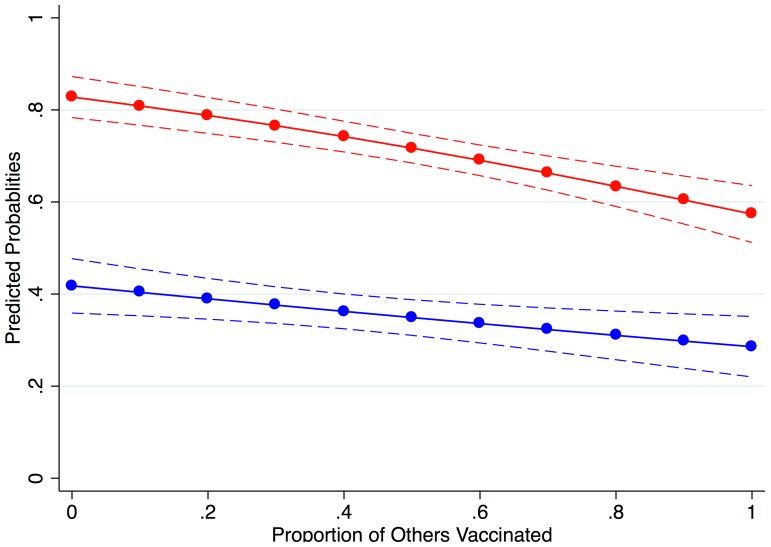
Predicted probability of vaccination and proportion of participants vaccinated, by risk condition. Red, high risk condition; and blue, low risk condition. Dashed lines show 95% confidence intervals. Multilevel logistic regression (Eq.1) was used to obtain the predicted probability of vaccination acceptance in high and low risk conditions. Observations from Round 2 to Round 24 were used for the analysis. The probability was evaluated with the mean values for the other independent variables except the proportion of others vaccinated.

We also performed sub-sample analyses by parameter condition to present the marginal effect of the proportion of others vaccinated on the probability of vaccination acceptance in each parameter condition. We grouped the observations by parameter condition, and analyzed the subgroups by the same logistic regression model used in the previous analysis (Table 5). The coefficient of the proportion of vaccination among others was significant in both parameter conditions in all the parameters, implying that a player's probability of vaccination lowered after observing higher vaccination coverage in the group in the previous round, regardless of parameter conditions. The predicted probability of vaccination acceptance, evaluated at mean values for other variables, was 83% for the high risk condition and 42% for the low risk condition when the proportion vaccinated in the previous round was 0%. According to our findings, the probability decreased with a wider vaccination rate: when the proportion vaccinated in the previous round was 100%, probability of vaccination in the current round was 57% and 29% for the high and low risk conditions respectively (Figure 3).

**Table 5:** Parameters and free-riding behavior: marginal effect of the proportion of vaccination in sub-sample analysis.

Player type		Cost of Vaccine		Risk of Infection		Severity of the influenza	
Elderly	−0.20 ^***^	Low	−0.24 ^***^	Low	−0.14 *	Mild	−0.30 ^***^
Young	−0.20^***^	High	−0.20 ^***^	High	−0.26 ^***^	Severe	−0.14^**^

* P<0.05,^ **^P<0.01, ^***^P<0.001, where the p-value was based on the coefficient estimates.

### Additional factors: influenza exposure or influenza prevalence

Two additional variables -influenza exposure and observed influenza prevalence among other group members - were added in order to assess the influence of two additional potential factors on vaccination decision-making. Player's influenza exposure showed a positive and statistically significant effect on vaccination (Table 6). On the other hand, the coefficient for the influenza prevalence was not statistically significant. In addition to the number of influenza exposure in the past during the game, two other variables were used to evaluate the impact of player's influenza exposure. One is the binary to represent if the player had been infected in the past during the game (Yes = 1), and the binary to represent if the player was infected during the previous round (Yes = 1). The result was robust when we used the two alternative measures for influenza exposure.

**Table 6:** Test for two additional determinants on vaccination decision-making: influenza prevalence and influenza exposure, results from multilevel logistic regressions.

	Reference group	Coefficient estimate		Standard error	Marginal effect	
Proportion of others vaccinated		−0.805^***^	0.20		−0.16	
Proportion of others infected		0.212	0.19		0.04	
Number of past infections		0.321^***^	0.05		0.07	
Intercept		−1.101^***^	0.21		N.A.	
Player type	Young	0.953^***^	0.13		0.21	
Cost of vaccine	High	0.582^***^	0.06		0.12	
Risk of infection	Low	1.628^***^	0.07		0.35	
Severity of the influenza	Mild	0.272^***^	0.06		0.06	
Number of rounds		−0.052^***^	0.01		−0.01	
Random effect (Player level) a		1.279	0.08		N.A.	

* P<0.05, ^**^P<0.01, ^***^P<0.001

a No formal statistical test was conducted to test if the random effect equals to zero.

Observations from Round 2 to Round 24 were used for the analysis. The random effect at the group level was not shown as it was estimated as zero.

## Discussion

This study examined an individual's vaccination decisions in the context of a simulated infectious disease characterized as influenza using the outcomes from a computerized interactive experimental game that mirrors the dynamic nature of infectious diseases. Due to the nature of infectious diseases, each individual's vaccination decision may depend on the vaccination decisions of other individuals. We found that as the proportion of vaccination among other group members increased, the likelihood of an individual choosing to get vaccinated in the following game round decreased, implying a free-riding motive in vaccination. This free-riding behavior was found regardless whether the influenza was severe or mild; the risk of infection was high or low; the cost of vaccine was high or low; and the participant played as an elderly or young person. Our results also indicated that the probability of vaccination acceptance increased with the exposure of a player to influenza during the game. There was no significant effect of influenza prevalence upon vaccination acceptance of an individual once vaccination rate was controlled for.

Previous studies on how a free-riding motive affects vaccination decision-making relied on hypothetical scenarios [7] [8]. We used computational laboratory experiments to exogenously control conditions regarding the disease and vaccination that may also affect an individual's vaccination choice. Our study is distinguished from these studies in at least two important aspects. First, it was based on an interactive laboratory experiment, allowing individuals' behavior to affect others' outcomes and subsequently influence any decisions made. The setting of the experiment enabled us to observe an individual's decision as the response to other individuals' decision. Second, our experiment brought an actual consequence to participants of the experimental game in the form of monetary payouts. In addition, our experimental approach can separate psychological factors such as beliefs or fears that are known to be determinants of vaccination in the real world from individuals' decision-making, and individuals weigh the expected cost and benefits of getting vaccinated given full information regarding diseases and vaccination.

It is well-documented that a change in perceived risk of infection with a disease alters individuals' behavior regarding prevention of the disease [18]. The free-riding behavior observed in our study could be interpreted in terms of a change in the perceived risk of infection. The probability of infection was a function of the number of those vaccinated. To make decisions, it appears that participants combined the risk condition controlled by the parameters in the experiment with the proportion of those who would get vaccinated, with the latter inferred from the vaccination coverage within the group in the previous round. Ultimately, the participants selected their choice based on the new perceived risk of infection. This is interesting as in each round the experiment ran independently and thus the perceived risk formed based on observations in the previous round had no direct influence on the objective risk of infection for a participant in the current round.

The free-riding behavior was observed regardless of parameter condition in the experiment. With the exception of the risk of infection parameter, the effects of the proportion of others vaccinated on the change in the predicted odds of vaccination acceptance did not differ across the parameter conditions. For instance, both young players and elderly players responded similarly to a change in the proportion of others vaccinated. Similarly, neither severity of influenza nor cost of vaccine mattered in free-riding behavior. Only the effects of the proportion of vaccination of others did vary with the risk of infection parameter, and was greater in high risk condition than in low risk condition. This result mirrored our assumption in the experiment that the reduction in the probability of infection with vaccination rate was larger in high risk condition than in low risk condition (See Table 1).

The finding that influenza exposure increased vaccination acceptance can also be interpreted in terms of changes in the individual's perceived risk of infection. Past disease exposure may increase individuals' perceptions of the risk of infection, which may in turn lead to an increase in the probability of vaccination acceptance. This finding has important implications regarding decision-making under uncertainty in the case of vaccination. Specifically, our results suggest that individuals can frequently update their perceived risk of infection based on their own experience and change their behavior to accommodate the new risk perception.

We found no significant effect of influenza prevalence on vaccination decision, which may seem at odds with previous empirical findings that indicate a significant impact of disease prevalence on demand for vaccination [19]. Indeed, we confirmed that the association between the probability of vaccination in the current round and influenza prevalence in the previous round was positive and statistically significant if we did not include vaccination rate in the previous round as an explanatory variable in the regression. In our experiment, the realized influenza prevalence was determined by two factors, the risk of infection parameter and the proportion of vaccination, and thus the vaccination rate and prevalence in the same round were not independent to each other. Because of the dependence between the two variables, the effect of influenza prevalence on vaccination decisions was partly mediated by vaccination rate in the previous round.

Previous studies have argued for the public policy implication of the impact of disease prevalence on vaccination decisions [20]. A similar scenario is applicable to the impact of vaccination coverage on vaccination acceptance. If fewer individuals are vaccinated because of observed higher vaccination coverage, a disease is given the opportunity to spread. Thus, if the observed high vaccination coverage provides a disincentive for individuals to get vaccinated, it becomes more difficult to enhance vaccination coverage in the community as vaccination coverage expands and therefore additional incentives may be needed to achieve higher vaccination coverage. Consistent with this argument, theoretical work has argued for prevalence-responsive subsidies, where the amount of subsidies is determined according to the disease prevalence [20]. The optimal policies, tax and/or subsidy, during a disease outbreak has been discussed [6]. In addition to these theoretical studies, a recent empirical study showed that a conditional cash transfer (CCT) program effectively improved vaccination coverage for selected vaccines for childhood diseases in Nicaragua [21].

Free-riding behavior has been intensively studied using experiments in the context of the provision of public goods. Our experiment was solely intended for the analysis of vaccination decisions, and cannot be taken as a direct parallel to those experiments on the public goods game. For example, in one of the standard models for the public good provision experiments, the linear public goods game [22] [23], choosing zero contribution regardless of other individuals' contribution is the theoretical prediction [22]. The free-riders that are predicted by a standard economic theory are defined as those who provide no contribution regardless of the mean contribution of others. In contrast, our analysis focused on an individual's contribution to the public (i.e. the individual's choice on vaccination) as the response to the decisions by other individuals in the group, and defined free-riding as decreased likelihood to be vaccinated in response to increased contribution of others.

In reality, the existence of peer may affect individual's decisions in other ways than free-riding behavior. For example, altruism could enhance vaccination uptake [7] [24]. Studies have argued bandwagoning effects and the role of imitations in vaccination decisions [7] [25], and peer effects through social network are also known factors to determine health behavior [26]. Also social norms or peer pressure could affect individual's choice in vaccination. This study focuses on the influence of free-riding on vaccination decisions among the effects of peer.

Through the analysis of individuals' decision-making about vaccination for a simulated infectious disease, we determined that an individual's vaccination decision was influenced by the vaccination status of others. Our results suggest that individual decisions may be driven by a free-riding motive as well as by prior influenza exposure. These factors should be taken into consideration by public policy makers in order to attain the necessary level of vaccination coverage.
