# Correction: Free-Riding Behavior in Vaccination Decisions: An Experimental Study

**DOI:** 10.1371/journal.pone.0094066

**Published:** 2014-03-27

**Authors:** The PLOS ONE Staff

The affiliation for the Editor, Maciej F. Boni, is incorrect. The correct affiliation is University of Oxford, Vietnam.
